# E-Learning in Teaching Emergency Disaster Response Among Undergraduate Medical Students in Malaysia

**DOI:** 10.3389/fpubh.2021.628178

**Published:** 2021-04-29

**Authors:** Ismail M. Saiboon, Fareena Zahari, Hisham M. Isa, Dazlin M. Sabardin, Colin E. Robertson

**Affiliations:** ^1^Department of Emergency Medicine, Faculty of Medicine, Universiti Kebangsaan Malaysia, Cheras, Malaysia; ^2^Accident and Emergency Medicine, University of Edinburgh, Edinburgh, United Kingdom

**Keywords:** education training, e-learning, disaster medicine, medical students, self-learning video

## Abstract

**Introduction:** Teaching disaster response medicine (DRM) to medical students requires considerable resources. We evaluate the effectiveness of e-learning in teaching emergency disaster response (ELITE-DR), a novel initiative, in educating medical students of the cognitive aspect of DRM.

**Methods:** A prospective cross-sectional study among pre-clinical year medical students was carried out to determine their knowledge on DRM and perception regarding the ELITE-DR initiative using a validated online questionnaire. A three-part self-learning video covering the principles and medical management of DRM were distributed before answering the questionnaire served as the training.

**Results:** A total of 168 students participated in the study. Their overall knowledge showed a significant increase in between pre-and-post-interventions. Recall and simple decision-making knowledge aspects were better than complex decision-making knowledge. It appeared that participants assimilate knowledge better from visual rather than audio stimuli. Participants with high perception-scores demonstrated better knowledge-scores. However, e-learning was not preferred as a substitute for face-to-face (F2F) teaching.

**Conclusion:** ELITE-DR shows promise in teaching DRM. Simple recall and comprehension levels of knowledge were well-served through this technique. However, for more complex decision-making knowledge, a different approach might be required. ELITE-DR offers flexibility, accessibility, and personalized learning. The content presentation is improved by using several different visual stimuli. This approach is useful for cognitive aspect learning, but it should not replace standard F2F teaching.

## Introduction

Globally, disaster medicine is inadequately represented in the undergraduate medical curriculum (1). Even though more medical schools have incorporated disaster medicine into their curriculum lately, it is believed that medical undergraduates still do not possess adequate knowledge and skill in this area (2). Teaching disaster response medicine (DRM) to undergraduate (UG) medical students is challenging, and not all medical schools include DRM as part of their curriculum (3–5). DRM teaching has many facets or components. Apart from knowledge, there are elements of planning, decision-making (both clinical and operative), simple or complex treatment strategies, clinical procedures, protocols, etc. (6–8). These are usually taught at the post-graduate level or as a subspecialty (9). However, as the recent COVID-19 pandemic has shown, UG education of disaster medicine is vital and should include some basic knowledge, simple procedures, and basic decision-making elements.

Disaster response is traditionally delivered using face-to-face (F2F) training, simulation, and role play (10, 11). This is a challenge for many academic institutions, as simulation role-play requires significant teaching manpower (12). Other difficulties include restrictions on teaching time within the 5- or 6-year curriculum (12, 13), difficulty gathering students in a single, suitable educational setting, and lack of direct access to experts (10).

In 2020, the COVID-19 pandemic has highlighted the difficulties in delivering UG curricula, especially for DRM (14–17). In Malaysia, as many parts of the world, this difficulty is brought about the government-imposed limitation to social interaction with the introduction of the Movement Control Order Act (18) that included suspending primary, secondary, and tertiary education. Since the majority of activities in DRM teaching involve gathering and close interactions, the implementation of the Movement Control Order Act posed a greater challenge to teaching DRM to UG medical students, as traditional methods of teaching become prohibited during the pandemic. Faced with these challenges, e-learning has been suggested as a promising alternative instead of traditional F2F methods (19–21).

In our institution, the Emergency Medicine module is taught in Year-5 over 1 credit hour (1 credit-hour = 40 notional hours). Presently DRM, which is part of this module, is taught in the form of face-to-face classroom method with field simulation exercise. This study attempts to experiment with deliverance of the cognitive aspect of teaching and learning contents of DRM as an online learning rather than face-to-face among the undergraduates in the pre-clinical years (Year-1 and Year-2), who are not normally students of this module. This study, e-learning in teaching emergency disaster response (ELITE DR), focused only on the cognitive aspect of DRM, uses a one-way asynchronous online video teaching. As outcomes of the study, we set out to evaluate the effectiveness of this novel initiative and the medical students' perception of it. The hypothesis was that the cognitive aspect of DRM could be taught effectively and acceptably using the ELITE-DR approach.

## Methodology

### Study Design

This was a prospective, cross-sectional, interventional study involving pre-clinical year students at Faculty of Medicine Universiti Kebangsaan Malaysia (UKM), looking at pre- and post-intervention outcomes. Enrolment for the study was performed using convenience sampling. Pre-clinical year medical students, from the first and second year of the program, were invited to participate in this study since DRM was not part of the pre-clinical year curriculum. Therefore, it removed the bias of doing this intervention by excluding students who had prior exposure to disaster management teaching or training. To further minimize potential confounders such as referring to other printed or online materials and discussion among the participants, it was emphasized to the participants that the marks in the questionnaires would not affect their curriculum assessment. This was to alleviate further stress among the participants and to promote compliance with the methodology. Each participant was required to watch a set of the self-learning-video (SLV) completely at least once.

The study was conducted between 2nd April 2019 and 31st March 2020. Approval was obtained from the Medical Research and Ethics Committee of UKM and funding was provided by the Faculty of Medicine as a Fundamental Research grant (approval number FF-2019-087).

### Development of the Self Learning Video (SLV)

**Figure 1:**
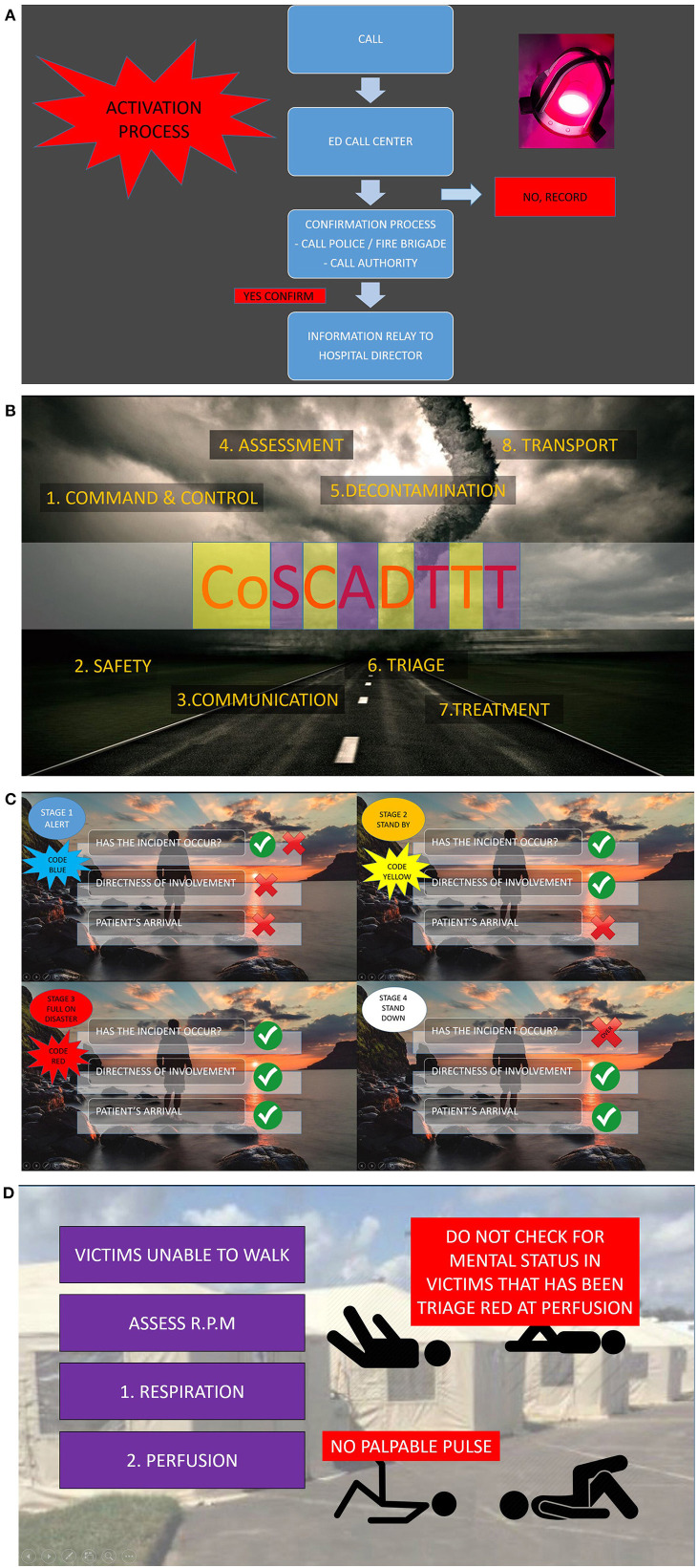
**(A–D)** Screen-shots from the ELITE-DR video. **(A)** Activation process of disaster response, **(B)** all-hazards principles of disaster response, **(C)** stages of disaster activation, and **(D)** triage process.

The SLV consisted of three video lectures of 8–10 min duration to maintain participants' focus and attention. The three videos covered the Principles of DRM and Disaster Medical management were covered. The video was developed using the Screencast-O-Matic application (version 0.2.2.3, Screencast-O-Matic, Seattle, WA, USA) as there were no available videos from the internet that adequately cover the principles of DRM. The Principles of DRM include definition, classification, phases, aims, activation process, and staging, while Disaster Medical management topics included: decontamination, triage, treatment, and transport. Screenshots from the videos showing the activation process, principles of disaster response (CoSCADTTT), disaster stages and triage are shown in Figures 1A–D.

The SLV content was validated by a panels of local emergency physicians who specialized in disaster medicine. They viewed the SLVs several times and made valuable remarks that contributed to the validity of the video. The recommended changes were made to the videos following comments from the panelists, and the process repeated until all panelists were satisfied with its content and arrangement. The study materials were then uploaded onto an online platform that was accessible to all participants.

### Questionnaire Development

The study collected quantitative data which included the participants' knowledge and perceptions. Respondents were invited to answer a self-administered questionnaire. Twenty questions assessed knowledge, while 26 questions assessed perceptions. Questions assessing knowledge were divided into principles and medical management of DRM. The knowledge-based questions were multiple-choice questions with a single best answer, and each question carried one (1) mark. The 26 items on the perceptions comprised the self-gain, presentability, and e-learning in the medical curriculum. These questions were developed by a group of three expert panels from among the local emergency physicians who specialized in DRM. The Delphi technique was used to develop the questionnaires. All questions were validated by expert panels of DRM to ensure those questions are clinically relevance. Further validation of the questionnaire was performed with a group of medical students from another institution that was not involved in this study.

Self-gain referred to the elements of that bring direct benefits to the participants and their preference, such as knowledge gained, flexibility, personalized learning, usefulness, ease of use, and familiarity of using online material or application in completing their task. In terms of presentability, the participants' preference for the SLV was evaluated. For e-learning in teaching DRM, those elements such as the use of e-learning as a standard teaching modality, substituting standard teaching with SLV, and recommendation of the SLV to fellow students were covered.

Perception was measured by using a 4-point Likert scale instrument. Participants were asked to rank their agreeability to the statements given based on a 4-graded scale. Marks were given for each scale from −2 for strongly disagree; −1 for disagree; +1 for agree; and +2 for strongly agree. The total score was then calculated and divided by the number of participants to give the perception score (PS). The mean PS score was used to categorize the perception of the participants ranging from strongly disagree to strongly agree according to the scale shown in Table 1.

**Table 1:** Perception's score (PS) scale.

**PS**	**Agreeability**
<-1 to −2	Strongly disagree
<0 to −1	Disagree
0 to +1	Agree
>+1 to +2	Strongly agree

### Study Protocol

The SLVs and the validated questionnaire were given to the eligible participants after briefing and consenting. A pre-test questionnaire was given to establish the baseline DRM knowledge among the participants. Each participant then received the three-part SLVs via web-links as below:

(Part 1) https://youtu.be/DAdVnFLozww
(Part 2) https://youtu.be/ySWylU-1xmI
(Part 3) https://youtu.be/3LiH0kS2brs.

The participants were given 7 consecutive days of access to the SLVs from the pre-test date. None of the participants received any teaching from facilitators during the learning session, as the intention was to simulate a self-instructional, unsupervised learning situation. Participants were strictly advised against referring to other resources of DRM either through printed materials or online or to discuss with each other in order to limit the potential confounding factors.

**Figure 2:**
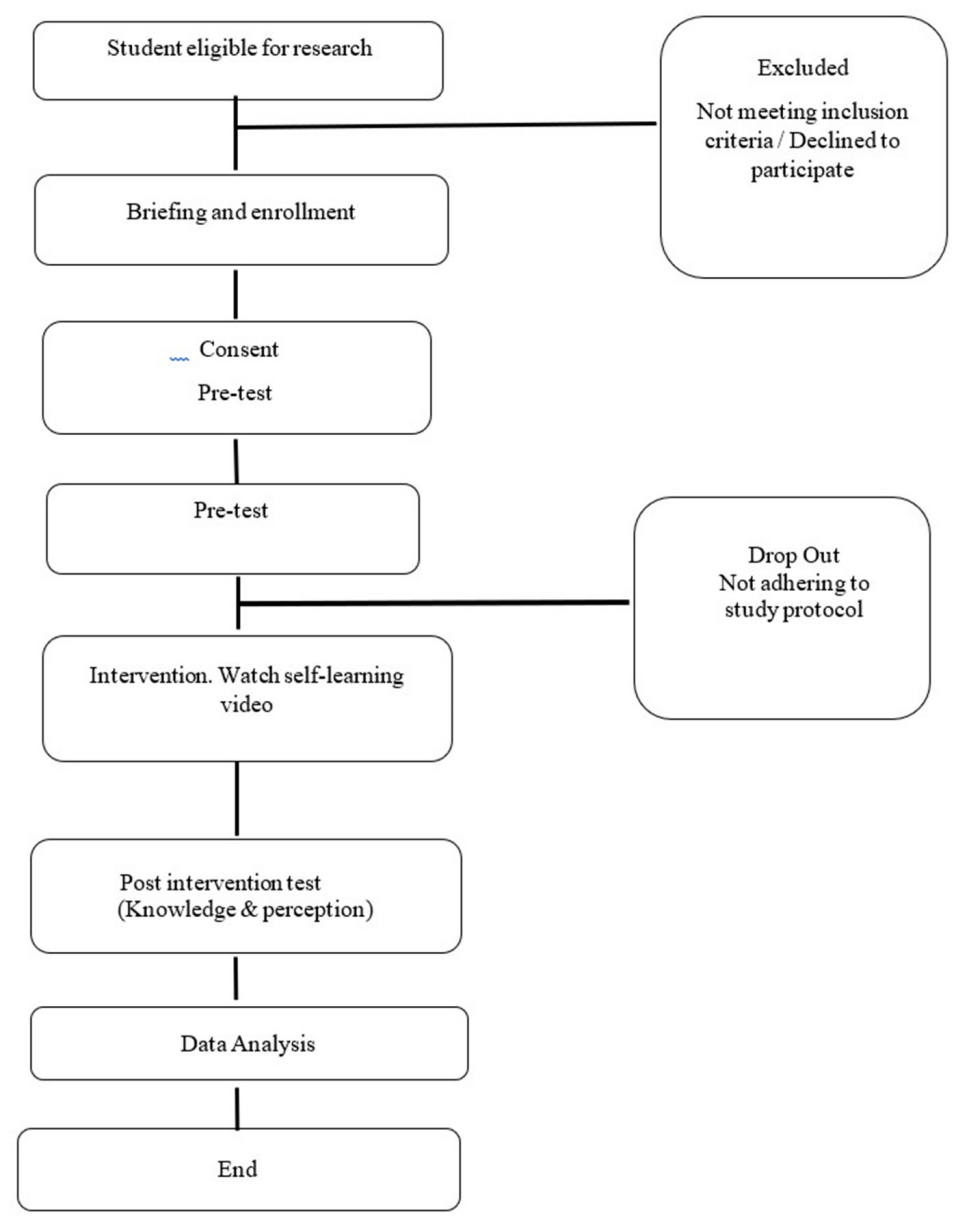
Study flow diagram.

Post-intervention assessment (post-test) on knowledge and perception was conducted using a self-administered questionnaire after the participants had completed their dedicated 7 consecutive days of learning. A flow diagram detailing the study is given in Figure 2.

### Statistical Analysis

There were 274 medical students in the pre-clinical years in the Faculty of Medicine UKM. Using the Krejcie and Morgan table (22), it was considered that a minimum of 155 participants was required to achieve a statistical power of 80% at a confidence interval of 95%. For the internal consistency measurement of the questionnaire, Cronbach's alpha score was 0.83.

Statistical analyses were performed using Minitab Statistical Software (version 19, Pennsylvania State University, PA, USA). Demographic characteristics of the participants were obtained by descriptive analysis. Data were summarized using mean and standard deviation for continuous variables, frequency, and percentages for categorical variables. The 99% confidence interval was calculated for the mean scores. A paired *t*-test was used to assess the mean differences between the two groups (knowledge between pre- and post-test), and one-way ANOVA was used to compare the PS achieve among the four groups of PS. All differences were considered statistically significant if *p* < 0.01.

## Results

**Figure 3:**
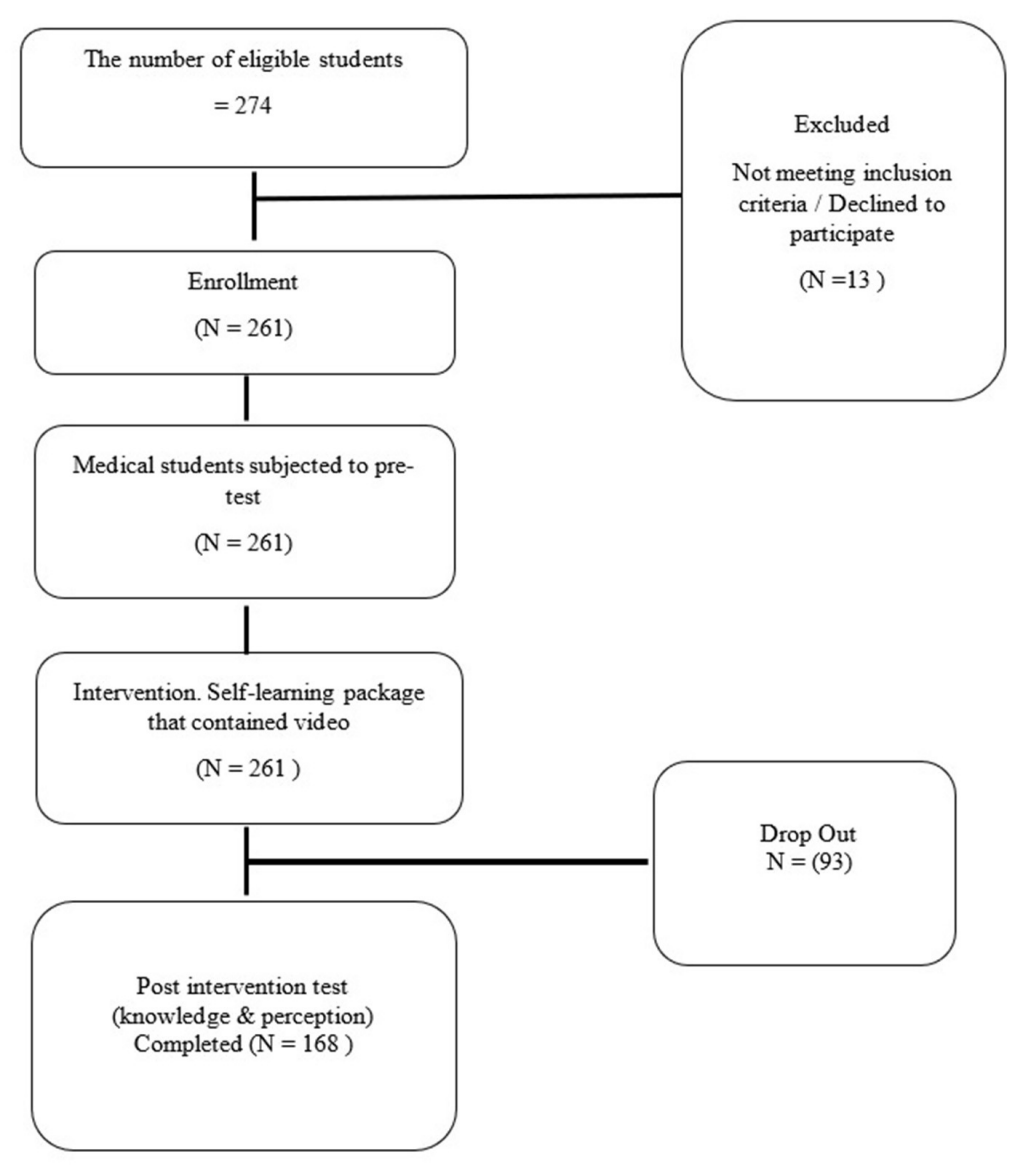
CONSORT flowchart of the study on E-Learning in Teaching Emergency Disaster Response (ELITE DR).

From a total of 274 undergraduate medical students in the pre-clinical year eligible for this study, 261 participants consented and were enrolled into the study. Thirteen were excluded from the study, 12 were because they had attended a disaster response medicine course prior this and one declined to participate. A total of 93 participants did not complete the study; a final total of 168 participants completed the study (Figure 3).

Of the 168 participants, the majority were females from the first year (*N* = 135; 80.4%). Most of the participants (86.3%) watched the SLVs only once. The demographic details of the participants are shown in Table 2.

**Table 2:** Demographic data of the participants.

**Demographic data of the participants**		
Gender	Frequency (*N* = 168)	Percentage (%)
Male	33	19.6
Female	135	80.4
**Year**
1	120	71.4
2	48	28.6
**Race**		
Malay	96	57.1
Chinese	30	17.9
Indian	25	14.9
Others	17	10.1
**Frequency of watching the SLP**		
1	145	86.3
2–4	22	13.1
>4	1	0.6

Table 3 shows the pre- and post-test data for knowledge on the principles, and medical management of DRM and their respective sub-components. The mean pre- and post-test marks for knowledge of DRM were 6.99 ± 2.65 and 13.31 ± 5.21, respectively (*p* < 0.001). On sub-analysis there was significant improvement in knowledge on the principles and medical management of DRM (*p* < 0.001).

**Table 3:** The mean score on pre-test and post-test for overall Knowledge, Principles, and Medical Management of Disaster Response.

**Knowledge scores**			
	**Pre-test** **(*N* = 168)**	**Post-test** **(*N* = 168)**	***p*****-value**
Knowledge on Disaster Response (overall)/20	6.99 ± 2.65	13.31 ± 5.21	<0.001
Knowledge on Principles of Disaster Response/9	2.91 ± 1.43	6.24 ± 2.53	<0.001
I. Definition II. Classification III. Phases IV. Stage V. Activation VI. Command and control VII. Safety	0.37 ± 0.48 0.1 ± 0.3 0.59 ± 0.49 1.1 ± 0.82 0.06 ± 0.23 0.26 ± 0.54 0.18 ± 0.38	0.76 ± 0.43 0.43 ± 0.5 0.88 ± 0.33 1.64 ± 0.68 0.67 ± 0.47 1.37 ± 0.79 0.68 ± 0.47	<0.001 <0.001 <0.001 <0.001 <0.001 <0.001 <0.001
Knowledge on Medical Management of Disaster Response/11	4.08 ± 1.97	7.06 ± 3.05	<0.001
I. Decontamination II. Triage III. Treatment IV. Transport	0.01 ± 0.08 2.78 ± 1.58 0.46 ± 0.49 0.58 ± 0.49	0.7 ± 0.46 5.39 ± 2.22 0.58 ± 0.49 0.65 ± 0.47	<0.001 <0.001 0.029 0.18

The medical management components like triage and decontamination also significantly improved between pre- and post-test (all *p* < 0.001) but not in the areas of treatment and transport with *p* = 0.029 and *p* = 0.180, respectively.

Table 4 shows the responses and mean score on perception for self-gain. Self-gain questions revealed that the participants “strongly agree” that e-learning is highly flexible, useful, easy to handle, and improved their knowledge. However, for familiarity with e-learning, such as finding the technique easier to use for revision, and allowing them to accomplish more work, the participants only scored “agree.”

**Table 4:** Result and mean score on perception for self-gain.

**Self-gain (*****N*** **= 168)**									
	**Strongly disagree** **(-2)**		**Disagree** **(−1)**		**Agree** **(+1)**		**Strongly agree** **(+2)**		**Mean PS** **(a + b + c + d)**/ **168**
	***n***	**a**	***n***	**b**	***n***	**c**	***n***	**d**	
Knowledge of disaster response has improved	1	−2	1	−1	95	+95	71	+142	+1.36
Content of the e-learning useful in my career	0	0	2	−2	95	+95	71	+142	+1.40
Able to watch at own pace	2	−4	6	−6	84	+84	76	+152	+1.35
I can learn at home, at work, at college, library or café (i.e., mobile)	2	−4	2	−2	81	+81	83	+166	+1.43
The knowledge presented in the e-learning is easy to understand	1	−2	2	−4	101	+101	62	+124	+1.31
Easier to revise electronic educational materials than printed material	7	−14	25	+25	95	+95	41	+82	+0.86
E-learning technologies allow accomplishing more work	4	−8	12	−12	117	+117	35	+70	+0.99
E-learning provides better learning opportunities (i.e.: accessibility to all without physical attendance)	3	−6	15	−15	101	+101	49	+98	+1.06
E-learning improves the quality of my studies	1	−2	4	−4	112	+112	51	+102	+1.24

*N = total number of respondents*.

*n = total respondent to each Likert Scale*.

*a = n × (−2); b = n × (−1); c = n × (+1); d = n × (+2)*.

*a, b, c, d = the total score for each category of Likert scale*.

Table 5 displays the result and mean score on SLV Presentability. The majority of the participants agreed or strongly agreed with the eye-catching visual stimuli of the videos (PS = +1.05 to +1.27). Animation, highlighted and enlarged words, and pictures were the preferred visual stimuli and helped participants retain facts (PS = +1.27). The participants scored “agree” (PS = +0.77 to +0.83) for audio stimuli, e.g., music, that had been incorporated in the videos.

**Table 5:** Result and mean score on SLV presentability.

**SLV presentability (*****N*** **= 168)**									
	**Strongly disagree** **(−2)**		**Disagree** **(−1)**		**Agree** **(+1)**		**Strongly agree** **(+2)**		**Mean PS** **(a + b + c + d)**/ **168**
	***n***	**a**	***n***	**b**	***n***	**c**	***n***	**d**	
The animation used able to sustain my focus	2	−4	11	−11	119	+119	36	+72	+1.05
The pictures used able to catch my attention	1	−2	11	−11	121	+121	35	+70	+1.06
The music helped to sustain my attention	2	−4	21	−21	126	+126	19	+38	+0.83
The music uplift my mood	3	−6	24	−24	123	+123	18	+36	+0.77
The bolded words attract my attention	2	−4	5	−5	105	+105	56	+112	+1.24
The enlarged words attract my attention	1	−2	3	−3	101	+101	63	+126	+1.26
The animation, pictures, and visual stimulus help me to remember the content better	2	−4	3	−3	105	+105	58	+116	+1.27

*N = total number of respondents*.

*n = total respondent to each Likert Scale*.

*a = n × (−2); b = n × (−1); c = n × (+1); d = n × (+2)*.

*a, b, c, d = the total score for each category of Likert scale*.

Table 6 shows the result and mean score on perception of the e-learning medical curriculum. Most of the participants would strongly recommend this video to others to enhance their learning ability of DRM (PS = +1.26 to +1.38). Most of the participants “agree” that DRM taught through e-learning, should be incorporated into the medical curriculum (PS = +0.71 to +0.99) in the form of adjunct material (PS = +1.12). However, they “disagree” (PS = −0.36) that DRM through e-learning could, or should, completely replace F2F sessions.

**Table 6:** Result and mean score on perception for e-learning in the medical curriculum.

**E-learning in the medical curriculum (*****N*** **= 168)**									
	**Strongly disagree** **(-2)**		**Disagree** **(−1)**		**Agree** **(+1)**		**Strongly agree** **(+2)**		**Mean PS** **(a + b + c + d)**/ **168**
	***n***	**a**	***n***	**b**	***n***	**c**	***n***	**d**	
Incorporate e-learning in the current medical curricular	2	−4	17	−17	110	+110	39	+78	+0.99
Medical curricular can be taught through e-learning	7	−14	31	−31	96	+96	34	+68	+0.71
E-learning should act as a supplementary to the F2F teaching	2	−4	9	−9	113	+113	44	+88	+1.12
E-learning substitute F2F lecture	42	−84	58	−58	55	+55	13	+26	−0.36
Recommend video to other medical students	1	−2	0	0	101	+101	66	+132	+1.38
Recommend the video for general public viewing	1	−2	6	−6	100	+100	61	+122	+1.27
Recommend this video to non-medical students	1	−2	6	−6	103	+103	58	+116	+1.26
Recommend this video to post-graduate students	1	−2	1	−1	109	+109	57	+114	+1.31
Recommend this video to general practitioners	1	−2	3	−3	107	+107	57	+114	+1.29
Would not recommend this video to be put on YouTube for free access	44	−88	68	−68	40	+40	16	+32	−0.5

*N = total number of respondents*.

*n = total respondent to each Likert Scale*.

*a = n × (−2); b = n × (−1); c = n × (+1); d = n × (+2)*.

*a, b, c, d = the total score for each category of Likert scale*.

Analysis of participants' knowledge and their PS revealed that those who graded higher PS could obtained a better knowledge score. One-way ANOVA analysis also revealed a significant difference between the PS groups' knowledge score (*p* < 0.001) as shown in Table 7.

**Table 7:** One-way ANOVA for the mean total score in four groups PS Strongly Agree, Agree, Disagree, and Strongly Disagree in Self-gain.

**Sample**	**Sample size**	**Total score mean**	**Standard** **deviation**	***p*-value**
Strongly agree	136	14.25	4.81	*p* < 0.001
Agree	30	11.77	5.33	
Disagree	2	7.5	0.71	
Strongly disagree	0	0	0	

## Discussion

This study has demonstrated that a novel e-learning package can successfully be used to teach DRM to UG medical students. Previous studies have been shown that e-learning can be as effective as F2F teaching when it comes to understanding knowledge aspects such as simple recall and comprehension (23–26). In contrast to the higher levels of learning outline in the Bloom's taxonomy, such as analysis, application, synthesis, and assessment, recall, and comprehension levels of learning are particularly well-served by e-learning. The participants, however, did not score well for medical management aspects, especially for the treatment and transport components. This was expected because the participants were pre-clinical students, and these subjects would have been unfamiliar to them where a deeper clinical understanding, further appraisal, and decision-making skills were required.

Feedback and debriefing have been shown to be important to enhance understanding in these areas (27). We may improve these skills if the feedback can be provided while viewing the video. An e-learning video platform that can assess the participants' understanding and collect their immediate feedback (28), such as ED-Puzzle or Common Ground (CG) scholar, may be one approach to this. The CG scholar platform has been shown to support critical thinking and promote a higher-order of thinking skills (29). It incorporates active knowledge production, ubiquitous learning, and recursive feedback, which involved receiving and giving reviews from peers and instructors to allow learners to reflect on their work. Our study used Screencast-O-Matic, a one-way directed video application that could not provide or create these higher-order thinking skills for the participants.

Participants did particularly well on the triage topic. This could be related to the structured algorithm, which was available in the video and hence required only simple decision-making (lower-order thinking). The structured algorithm directed the participants to follow the signs or symptoms elicited before deciding.

Perceptions were categorized according to the PS scale in Table 1. In self-gain, the participants agreed on most of the learning items, especially those involving knowledge improvement, flexibility, personalization, usefulness, and ease of handling. They found e-learning easier to use and convenient. Having a flexible and personalized e-learning environment is important to encourage an active and inclusive learning environment (30). However, in terms of familiarity, such as revising using digital materials alone or using technologies in accomplishing more work, the PS score was much lower. Therefore, while participants find e-learning helpful, they need to be familiarized with e-learning tools, especially when searching for new data or knowledge.

Limitations in the familiarity with usage of digital technology can lead to difficulties for students to accomplish their goals. Consequently, the effort they need to invest to generate the expected outcomes tends to be higher than that of students better familiarized with online technologies (31). In terms of presentability, the participants awarded high scores for visual inputs compared to audio inputs. This is coherent with a previous study where visual computer-based learning did not show any significant difference with or without an auditory narrative (32). It appeared that visual stimuli were preferred to audio stimuli for factual learning and knowledge retention.

In terms of e-learning incorporation in the DRM, the participants agreed with most of the statements, except the statement that e-learning could replace F2F teaching totally. Presumably, this is because, in F2F teaching, students receive not just visual stimuli but also audio, emotional, tactile, and feedback stimuli that make a more immersive, powerful, and retentive learning experience. Furthermore, psychomotor skills could not be adequately covered with e-learning. However, participants agreed that e-learning is greatly beneficial, but not up to the level of replacing the current format of DRM teaching.

It is interesting to note that most of our participants in this study were females as shown in Table 2. This is generally due to there are more female students in medical school throughout Malaysia with a ratio of about 60:40 (female:male) (33). Another reason why there were more female participants was due to larger dropout rate among male participants (48%, *n* = 30) as compared to females (36%, *n* = 76) who did not complete the study.

## Study Limitations

The number of questions in the questionnaires is relatively low and might not be adequate in assessing some topics. Nevertheless, our study gives a general idea of the capability of video teaching in acquiring factual knowledge and decision making in DRM. Future studies may target decision-making topics with more emphasis on assessing higher-order thinking skills.

This study did not explore or evaluate the psychomotor skills that students would usually perform during F2F simulation exercises of DRM, including Airway, Breathing, Circulation, and Immobilization procedures. These include standard first aid procedures (bleeding and wound management, splinting, and bandaging), invasive procedural skills (airway and ventilation management, intravenous cannulation), carrying and lifting of victims, etc. Generally, an online platform always has difficulties in teaching and assessing procedural skills. A further study in assessing teaching and learning procedural skills could be conducted in the future.

We did not evaluate the relative time spent by the participants in watching the three videos. This might help reveal whether the duration of the video watching relates to the effectiveness of learning and knowledge retention.

The videos in our study do not have “closed-captioning” since we felt that all the important facts have been spelled out in the videos. However, having closed captioning might have had positive influence on the participants' understanding. This will constitute further improvement in our future educational material or research using video for e-learning. Another limitation of our video was the lack of features for printing key points or summary from the video. Providing the viewers the ability to print key points or a summary of the videos might help in learning. This feature allows key frames of the segment to be converted to textual annotation using segmental encoding and decoding deep learning models (34).

The study also did not compare synchronous (real-time interaction using any online application like Zoom, Microsoft Teams, etc.) with asynchronous teaching (not real-time interaction) of DRM. This study employed an asynchronous one-way video teaching rather than synchronous e-learning with feedback incorporated. Future research should explore the impact of synchronous and asynchronous e-learning with and without incorporated feedback to determine the best way of delivering DRM to UG medical students.

Our study did not compare with the other technique of disaster response medicine teaching because most of the time this subject were taught using a classroom medium either as a face-to-face immersive simulation, table-top exercise or hybrid simulation of approach combining e-learning with classroom immersive simulation. Therefore, we felt it was not appropriate to compare our study that utilize a full asynchronous online approach with the current established methods. Furthermore, we did not evaluate the psychomotor skills in our study.

Finally, this study did not incorporate debriefing even though debriefing is a very powerful learning tool because debriefing was not suitable for an asynchronous methods of teaching. Furthermore, debriefing after watching the video would disrupt the findings of the study. We wanted to determine the effect of a pure asynchronous online teaching on disaster response medicine topics. However, in the future study, we are going to include the element of debriefing with the synchronous mode of teaching.

## Conclusion

Our study revealed that ELITE-DR, a novel e-learning platform is beneficial in teaching-learning of emergency DRM among UG medical students. Recall of knowledge comprehension and simple analysis-application for basic decision-making was particularly well-served through ELITE-DR, whereas complex decision-making knowledge aspects such as treatment and transport decisions were likely to require a different approach, perhaps one that incorporates feedback. Higher flexibility, usefulness, ease of access, and personalized learning were some of the benefits that make e-learning an acceptable approach for DRM teaching. In e-learning, UG students preferred visual stimuli compared to audio stimuli. The majority of UG students agreed that e-learning could provide as an adjunct but should not replace F2F teaching.

## Data Availability Statement

The raw data supporting the conclusions of this article will be made available by the authors, without undue reservation.

## Ethics Statement

The studies involving human participants were reviewed and approved by Medical Research and Ethics Committee of Universiti Kebangsaan Malaysia. The patients/participants provided their written informed consent to participate in this study.

## Author Contributions

IS, FZ, HI, DS, and CR provided the study concept and performed data analysis. FZ and IS performed data collection. IS, FZ, and CR interpreted the data. All authors were involved in writing and critically reviewing the final manuscript.

## Conflict of Interest

The authors declare that the research was conducted in the absence of any commercial or financial relationships that could be construed as a potential conflict of interest.

## Abbreviations

DRM: disaster response medicine
ELITE DR: e-learning in teaching emergency disaster response
F2F: face-to-face
PS: perception score
SLV: self-learning-video
UG: undergraduate.

## References

[1] Kasselmann N Bickelmayer J Peters H Wesemann U Oestmann JW Willy C. Relevance of disaster and deployment medicine for medical students: a pilot study based on an interdisciplinary lecture series. Unfallchirurg. (2020) 123:464–72. 10.1007/s00113-019-00738-w 31696247

[2] Tsai YD Tsai SH Chen SJ Chen YC Wang JC Hsu CC. Pilot study of a longitudinal integrated disaster and military medicine education program for undergraduate medical students. Medicine. (2020). 99:e20230 10.1097/MD.0000000000020230 32443354 PMC7461121

[3] Sinha A Pal DK Kasar PK Tiwari R Sharma A. Knowledge, attitude and practice of disaster preparedness and mitigation among medical students. Disaster Prev Manag An Int J. (2008) 17:503–7. 10.1108/09653560810901746

[4] Saiboon IM Jaafar JM Nasarudin NMA Md Jamal S Ahmad Z Harunarashid H. Development of web-based learning packages for emergency skills. Proc Soc Behav Sci. (2012) 60:426–9. 10.1016/j.sbspro.2012.09.401

[5] Alexander AJ Bandiera GW Mazurik L. A multiphase disaster training exercise for emergency medicine residents: opportunity knocks. Acad Emerg Med. (2005) 12:404–9. 10.1111/j.1553-2712.2005.tb01538.x 15860693

[6] Bajow N Djalali A Ingrassia PL Ageely H Bani I Corte FD. Proposal for a community-based disaster management curriculum for medical school undergraduates in Saudi Arabia. Am J Disaster Med. (2015). 10:145–52. 10.5055/ajdm.2015.0197 26312495

[7] Back DA Lembke V Fellmer F Kaiser D Kasselmann N Bickelmayer J. Deployment and disaster medicine in an undergraduate teaching module. Mil Med. (2019). 184:e284–9. 10.1093/milmed/usy250 30281084

[8] Saiboon IM Jaafar MJ Harunarashid H Jamal SM. The effectiveness of simulation based medical education in teaching concepts of major incident response. Proc Soc Behav Sci. (2011) 18:372–8. 10.1016/j.sbspro.2011.05.053

[9] Algaali KY Djalali A Della Corte F Ismail MA Ingrassia PL. Postgraduate education in disaster health and medicine. Front Public Health. (2015) 3:185. 10.3389/fpubh.2015.00185 26322298 PMC4530259

[10] Curtis HA Trang K Chason KW Biddinger PD. Video-based learning vs. traditional lecture for instructing emergency medicine residents in disaster medicine principles of mass triage, decontamination, and personal protective equipment. Prehosp Disaster Med. (2018) 33:7–12. 10.1017/S1049023X1700718X 29317001

[11] Wiesner L Kappler S Shuster A DeLuca M Ott J Glasser E. Disaster training in 24 hours: evaluation of a novel medical student curriculum in disaster medicine. J Emerg Med. (2018) 54:348–53. 10.1016/j.jemermed.2017.12.008 29395693

[12] Saiboon IM Jaafar MJ Ahmad NS Ahmad Z Hamzah FA Jamal SM. Simulation based education in delivering emergency medicine module. Proced Soc Behav Sci. (2011) 18:388–93. 10.1016/j.sbspro.2011.05.056

[13] Bajow N Djalali A Ingrassia PL Ragazzoni L Ageely H Bani I. Evaluation of a new community-based curriculum in disaster medicine for undergraduates. BMC Med Educ. (2016) 16:225. 10.1186/s12909-016-0746-6 27562428 PMC5000399

[14] McCloskey B Zumla A Ippolito G Blumberg L Arbon P Cicero A. Mass gathering events and reducing further global spread of COVID-19: a political and public health dilemma. Lancet. (2020) 395:1096–9. 10.1016/S0140-6736(20)30681-4 32203693 PMC7138150

[15] Rose S. Medical student education in the time of COVID-19. JAMA. (2020) 10.1001/jama.2020.5227 32232420

[16] Chatterjee Surobhi. The COVID-19 pandemic through the lens of a medical student in India. Int J Med Stud. (2020) 8:82–3. 10.5195/ijms.2020.537

[17] Ruthberg JS Quereshy HA Ahmadmehrabi S Trudeau S Chaudry E Hair B. A multimodal multi-institutional solution to remote medical student education for otolaryngology during COVID-19. Otolaryngol Head Neck Surg. (2020) 163:707–9. 10.1177/0194599820933599 32515642

[18] Tang A. (2020). Malaysia Announces Movement Control Order After Spike in Covid-19 Cases (Updated). The Star. Available online at: https://www.thestar.com.my/news/nation/2020/03/16/malaysia-announces-restricted-movement-measure-after-spike-in-covid-19-cases (accessed June 18, 2020).

[19] Cardall S Krupat E Ulrich M. Live lecture versus video-recorded lecture: are students voting with their feet? Acad Med. (2008) 83:1174–8. 10.1097/ACM.0b013e31818c6902 19202495

[20] Debacker M Delooz H Corte F Della. The European master program in disaster medicine. Int J Disaster Med. (2003) 1:35–41. 10.1080/15031430310004230

[21] Zaini NA Noor SFM Zailani SZM. Design and development of flood disaster game-based learning based on learning domain. Int J Eng Adv Technol. (2020) 9:679–85. 10.35940/ijeat.C6216.049420

[22] Krejcie R V Morgan DW. Determining sample size for research activities. Educ Psychol Meas. (1970) 30:607 10.1177/001316447003000308

[23] Salmon G Tombs M Surman K. Teaching medical students about attention deficit hyperactivity disorder (ADHD): the design and development of an e-learning resource. Adv Med Educ Pract. (2019) 10:987–97. 10.2147/AMEP.S220390 31819698 PMC6876212

[24] McGready J Brookmeyer R. Evaluation of student outcomes in online vs. campus biostatistics education in a graduate school of public health. Prev Med. (2013) 56:142–4. 10.1016/j.ypmed.2012.11.020 23219758

[25] Worm BS. Learning from simple ebooks, online cases or classroom teaching when acquiring complex knowledge. A randomized controlled trial in respiratory physiology and pulmonology. PLoS ONE. (2013) 8:e73336. 10.1371/journal.pone.0073336 24039917 PMC3767787

[26] Saiboon IM Qamruddin RM Jaafar JM Bakar AA Hamzah FA Eng HS. Effectiveness of teaching automated external defibrillators use using a traditional classroom instruction versus self-instruction video in non-critical care nurses. Saudi Med J. (2016) 37:429–35. 10.15537/smj.2016.4.14833 27052286 PMC4852021

[27] Donkin R Askew E Stevenson H. Video feedback and e-Learning enhances laboratory skills and engagement in medical laboratory science students. BMC Med Educ. (2019) 19:310. 10.1186/s12909-019-1745-1 31412864 PMC6693214

[28] Haniya S Montebello M Cope B Tapping R. Promoting critical clinical thinking through e-learning. In: Proceedings of the 10th International Conference on Education and New Learning Technologies. Palma: EduLearn18 (2018). 10.21125/edulearn.2018.1235

[29] de Leng BA Dolmans DHJM Jöbsis R Muijtjens AMM van der Vleuten CPM. Exploration of an e-learning model to foster critical thinking on basic science concepts during work placements. Comput Educ. (2009) 53:1–13. 10.1016/j.compedu.2008.12.012

[30] Nganji JT. Towards learner-constructed e-learning environments for effective personal learning experiences. Behav Inf Technol. (2018) 37:647–57. 10.1080/0144929X.2018.1470673

[31] Moreno V Cavazotte F Alves I. Explaining university students' effective use of e-learning platforms. Br J Educ Technol. (2017) 48:995–1009. 10.1111/bjet.12469

[32] Mounsey A Reid A. Auditory narrative does not improve learning when added to visual computer-based learning modules. Med Sci Educ. (2019) 29:1043–9. 10.1007/s40670-019-00801-6 PMC8368538 34457582

[33] Myint YY Tun Y. Gender ratio in undergraduate medical program, Medical Faculty, IIUM. In: IRIIE 2010. Kuala Lumpur: International Islamic University of Malaysia (2010). Available online at: http://irep.iium.edu.my/10878/ (accessed April 12, 2021).

[34] Sah S Kulhare S Gray A Venugopalan S Prud'Hommeaux E Ptucha R. Semantic text summarization of long videos. In: 2017 IEEE Winter Conference on Applications of Computer Vision (WACV). Santa Rosa, CA (2017). p. 989–97. 10.1109/WACV.2017.115

